# Generalized Bullous Fixed Drug Eruption Secondary to Levocetirizine: A Lesson Learned!

**DOI:** 10.1002/ccr3.71417

**Published:** 2025-11-04

**Authors:** Mahesh Mathur, Sumit Paudel, Nabita Bhattarai, Sambidha Karki, Sandhya Regmi

**Affiliations:** ^1^ Department of Dermatology College of Medical Sciences Teaching Hospital Bharatpur Nepal

**Keywords:** antihistamine, fixed drug eruption, generalized bullous fixed drug eruption, levocetirizine, patch test

## Abstract

Fixed drug eruption (FDE) is a unique type of adverse drug eruption characterized by a well‐defined erythematous to violaceous patch that recurs at the same site upon re‐exposure to causative drugs and resolves with hyperpigmentation. This distinct drug reaction is a type IV hypersensitivity reaction that is mediated by memory CD8+ T cells that reside in the basal layer of the epidermis of resting FDE lesions. Generalized bullous FDE (GBFDE) is the most severe form of FDE, presenting with widespread blisters and erosions that can be misdiagnosed as epidermal necrolysis. There are only two reports of levocetirizine‐induced GBFDE in the literature to date. We hereby present a case of a 65‐year‐old male with GBFDE due to levocetirizine, which was misattributed to a more commonly implicated drug, that is, acetaminophen.

**Abbreviations**
FDE: fixed drug eruption
GBFDE: generalized bullous FDE
SJS/TEN: Steven–Johnson syndrome/toxic epidermal necrolysis

**Summary**

- This case highlights generalized bullous fixed drug eruption caused by levocetirizine, initially misattributed to the more commonly implicated acetaminophen.
- Recurrent, site‐specific lesions with increasing severity emphasize the need for accurate drug identification as prompt recognition and avoidance of the culprit drug can prevent potentially serious adverse reactions.

## 1 Introduction

Fixed drug eruption (FDE) is a unique type of adverse drug eruption characterized by a well‐defined erythematous to violaceous patch that recurs at the same site upon re‐exposure to causative drugs and resolves with hyperpigmentation [1, 2]. Generalized bullous FDE (GBFDE) is the most severe form of FDE, presenting with widespread blisters and erosions that can be misdiagnosed as epidermal necrolysis [1]. Levocetirizine, despite being one of the most common antihistamines used, rarely causes cutaneous reactions [3]. In this report, we describe a case of GBFDE associated with levocetirizine.

## 2 Case Presentation

**FIGURE 1:**
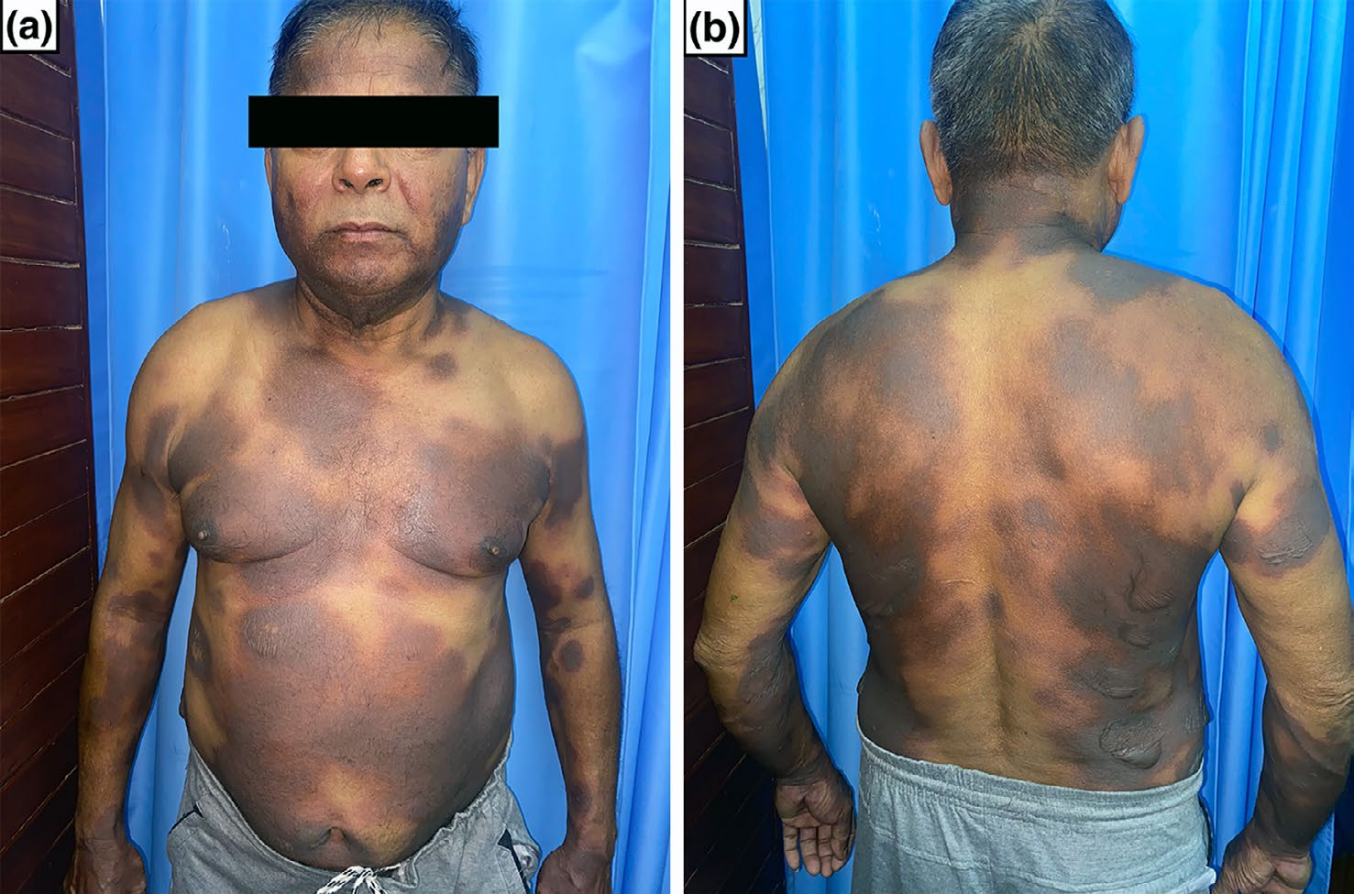
Multiple well‐defined, dusky, violaceous to erythematous patches, and painful bullae on the face, anterior trunk, posterior trunk, and bilateral upper extremities (a, b).

A 65‐year‐old male presented with multiple well‐defined, dusky, violaceous to erythematous patches and painful bullae on the face, anterior and posterior trunk, bilateral upper and lower extremities for 2 days (Figure 1a,b). He also had crusting over the upper lip along with erosions over the prepuce. He took levocetirizine 2 days back for allergic rhinitis and within a few hours of intake of the drug, the skin lesions developed. There is a past history of two episodes of similar lesions on taking levocetirizine and acetaminophen, the severity of the reaction increasing with each subsequent exposure. The previously involved areas were affected in addition to prior uninvolved sites. In the last two episodes, the probable offending drug was thought to be acetaminophen, so the patient was advised to avoid acetaminophen only. Patch testing and oral rechallenge tests were not performed in the last two episodes. In the current episode, the patient only took levocetirizine; this suggests the drug reaction is secondary to levocetirizine.

## 3 Differential Diagnosis, Investigations, and Treatment

**FIGURE 2:**
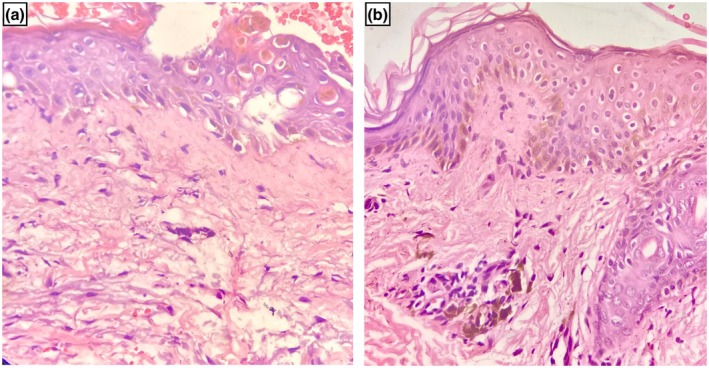
Hematoxylin and eosin staining (40×) showed basal cell vacoulization (blue arrow), apoptotic keratinocytes (black arrow), melanin incontinence (red arrow), and perivascular inflammatory infiltrates (green arrow) (a, b).

The patient was hospitalized, and generalized bullous fixed drug eruption (GBFDE) and Steven–Johnson syndrome/toxic epidermal necrolysis (SJS/TEN) were kept as differential diagnoses. Routine blood investigations were within normal limits. Blood gas analysis was normal. Skin biopsy showed basal cell vacuolization, apoptotic keratinocytes, melanin incontinence in the upper dermis, and perivascular inflammatory infiltrates (Figure 2a,b). The Naranjo adverse drug reaction probability scale was calculated to be 10, which indicates a definite adverse drug reaction to levocetirizine. Patch testing was not performed as it was not available at our center. Oral rechallenge test was avoided due to the severity of the reaction. As there was a history of similar lesions in the past, latency between drug intake and lesion onset was a few hours, no systemic symptoms, blood investigations were normal and histopathological findings were of interface dermatitis without extensive epidermal necrosis, diagnosis of GBFDE was made and he was started on oral prednisolone (0.5 mg/kg day). The vesicles and bullae settled after 10 days, leaving dusky brown residual hyperpigmentation. Oral prednisolone was discontinued after 2 weeks. The patient was advised to avoid levocetirizine and other piperazine antihistamines.

## 4 Conclusion and Results

We referred him to another center for patch testing to confirm the diagnosis and find cross‐reactivity with other groups of antihistamines so as to prevent such episodes in the future.

## 5 Discussion

FDE is characterized by same‐site recurrence with an increase in size and number of lesions on additional exposure to the offending medication [1, 2]. This distinct drug eruption is a type IV hypersensitivity reaction that is mediated by memory CD8+ T cells that reside in the basal layer of the epidermis of resting FDE lesion [1, 2]. Several presentations of FDE described in the literature are pigmented, bullous, generalized, generalized bullous, linear, non‐pigmenting, mucosal, wandering, and erythema multiforme‐like [1, 4]. GBFDE is a rare and severe variant of FDE with blisters and erosions involving at least 10% body surface area on at least 3 of 6 anatomical sites, which are head/neck, anterior, posterior trunk, upper and lower extremities, and genitalia [1]. The recurrence of FDE is associated with increased inflammation and hyperpigmentation with each occurrence, ultimately leading to GBFDE [1].

The drugs most commonly implicated in FDE are acetaminophen, non‐steroidal anti‐inflammatory drugs, anticonvulsants, and antibiotics [1, 2]. The antihistamines are the commonly used drugs for supportive management of adverse drug reactions; however, they can rarely cause FDE. Among the piperazine group, hydroxyzine, buclizine, cyclizine, and meclizine are the first‐generation antihistamines, and cetirizine and levocetirizine are the second‐generation antihistamines. Piperidine derivatives include first‐generation antihistamines such as azatadine, cyproheptadine, diphenylpyraline, and ketotifen, and second‐generation ones include astemizole, loratadine, desloratadine, ebastine, fexofenadine, olopatadine, and terfenadine [5]. Levocetirizine, a piperazine derivative, is a second‐generation antihistamine commonly prescribed in dermatological practice. Despite being a safe drug, it can infrequently cause cutaneous side effects, including pruritus, urticaria, angioneurotic edema and FDE [3]. In the literature, only two cases of GBFDE due to levocetirizine have been reported to date [6]. The cross‐reactivity of levocetirizine with other piperazine antihistamines like cetirizine and hydroxyzine has been described [5, 6].

FDE can be diagnosed on clinical grounds; however, skin biopsy, topical patch test, and systemic rechallenge may help to confirm the diagnosis [1, 2]. The systemic rechallenge test is contraindicated in both GBFDE and EN due to the severity of the reaction. A patch test is considered safe but less sensitive, with response rates varying among different studies from 33% to 80% [7]. In cases of FDE or GBFDE, patch testing is preferably done on previously affected hyperpigmented skin, if the site is accessible; otherwise, it can be performed as usual on the patient's back [7, 8]. This test is usually not necessary in a patient with a clear recent history of FDE following exposure to only one single drug agent, where the drug is a known common cause of FDE; however, it is helpful where multiple culprits were involved, cross‐reactive alternatives are needed, or where there is diagnostic doubt [8].

The generalized distribution with skin detachment seen in GBFDE can easily be misdiagnosed as EN [7]. GBFDE and EN are two conditions with differences in clinical features, history of previous similar reactions, latency period between the beginning of drug use and reaction onset, and underlying pathogenesis [7, 9]. Even though GBFDE has a considerably better prognosis than SJS/TEN, it is still potentially life‐threatening and deserves the same care and supportive treatment as EN [1, 9]. Treatment involves avoidance of the causative drug along with topical or oral steroids, cyclosporine, and admission to the burn unit in severe cases for wound care [1, 9].

In our patient, GBFDE due to levocetirizine was misattributed to a more commonly implicated drug, that is, acetaminophen which led to a delay in diagnosis and increased severity of the reaction. So this case is being reported to enlighten the dermatologists about a severe adverse drug reaction induced by a drug prescribed routinely, which will aid in early diagnosis and prompt management.

## Author Contributions

**Mahesh Mathur:** conceptualization, formal analysis, resources, supervision, validation, visualization, writing – original draft. **Sumit Paudel:** conceptualization, formal analysis, resources, supervision, validation, visualization. **Sambidha Karki:** data curation, investigation, visualization, writing – review and editing. **Nabita Bhattarai:** data curation, investigation, visualization, writing – review and editing. **Sandhya Regmi:** conceptualization, formal analysis, resources, supervision, validation, visualization, writing – original draft.

## Ethics Statement

This study was reviewed and approved by the Institutional Review Board College of Medical Sciences (IRBCOMS).

## Consent

The patient in this manuscript has given written informed consent to the publication of his case details.

## Conflicts of Interest

The authors declare no conflicts of interest.

## Data Availability

The data that support the findings of this study are available from the corresponding author upon reasonable request.
